# Entorhinal cortex atrophy mediates the association of plasma p-tau181, GFAP, and NfL with cognitive impairment in Parkinson’s disease

**DOI:** 10.3389/fnagi.2026.1791756

**Published:** 2026-05-08

**Authors:** Qiangqiang Wan, Xinyu Dai, Zhongwen Zhang, Ping Shan, Gui Mei, Luqi Huang, Lan Wang, Hongfen Peng

**Affiliations:** 1 Department of Laboratory Medicine, Wuhan No. 1 Hospital, Wuhan, China; 2 Engineering Research Center of TCM Protection Technology and New Product Development for the Elderly Brain Health, Ministry of Education, Hubei University of Chinese Medicine, Wuhan, China; 3 Department of Neurology, Traditional Chinese and Western Medicine Hospital of Wuhan, Tongji Medical College, Huazhong University of Science and Technology, Wuhan, China; 4 Hubei Provincial Hospital of Traditional Chinese Medicine, Wuhan, China; 5 Department of Radiology, Traditional Chinese and Western Medicine Hospital of Wuhan, Tongji Medical College, Huazhong University of Science and Technology, Wuhan, China

**Keywords:** cognitive impairment, entorhinal cortex, neurofilament light chain, Parkinson’s disease, plasma biomarkers

## Abstract

**Background:**

Cognitive impairment is a major determinant of disability in Parkinson’s disease (PD), underscoring the need for biomarkers that reflect its underlying pathophysiology. Plasma biomarkers, including phosphorylated tau at threonine 181 (p-tau181), glial fibrillary acidic protein (GFAP), and neurofilament light chain (NfL), are promising candidates, yet their independent predictive value and the pathways by which they relate to cognitive function in PD remain unclear.

**Methods:**

We enrolled 89 PD patients without dementia (37 with normal cognition and 52 with mild cognitive impairment) and 40 healthy controls. Plasma biomarkers were measured using single-molecule array technology. All participants underwent high-resolution structural magnetic resonance imaging and cognitive assessment with the Montreal Cognitive Assessment (MoCA). Hierarchical regression evaluated the independent contribution of each biomarker to MoCA scores. Mediation and moderated mediation analyses were then conducted to test whether atrophy in brain regions of interest mediates the effects of biomarkers on cognitive function and whether these pathways were moderated by cognitive status.

**Results:**

Plasma p-tau181, GFAP, and NfL were each independently associated with worse global cognition. The effects of all three biomarkers were significantly and specifically mediated by atrophy of the entorhinal cortex (ERC), but not by other tested regions. Critically, the mediation pathway for NfL was specifically moderated by cognitive status, being significant only in PD patients with mild cognitive impairment.

**Conclusion:**

The associations of plasma p-tau181, GFAP, and NfL on cognitive impairment in PD converges on ERC atrophy. The association between NfL and cognition via ERC atrophy is dynamically potentiated at the mild cognitive impairment stage, positioning NfL as a stage-specific biomarker. An integrative model combining these plasma biomarkers with ERC integrity could enhance risk stratification for cognitive impairment in PD.

## 1 Introduction

Parkinson’s disease (PD) is the second most common neurodegenerative disorder worldwide, with cognitive impairment emerging as a frequent and highly disabling non-motor symptom (Savica et al., 2018; Ben-Shlomo et al., 2024). The pathological underpinnings of cognitive impairment in PD are complex, and its progression is heterogeneous, presenting a significant challenge for prognosis (Aarsland et al., 2021). This underscores a critical need for robust biomarkers to identify at-risk individuals and track cognitive decline.

Advances in ultrasensitive assays have enabled the reliable measurement of neurodegeneration- and glial activation-related proteins in blood plasma. Phosphorylated tau at threonine 181 (p-tau181), glial fibrillary acidic protein (GFAP), and neurofilament light chain (NfL)–well-validated biomarkers in Alzheimer’s disease (Frank et al., 2022; Brickman et al., 2021; Guo et al., 2024; Pelkmans et al., 2024) – are now being explored in synucleinopathies like PD and dementia with Lewy bodies (Pilotto et al., 2024; Donaghy et al., 2025; Raghavan et al., 2025). These markers show promise in predicting clinical progression (Lee et al., 2025; Giacomucci et al., 2022; Mazzeo et al., 2024; Chen et al., 2026). However, within the context of PD-related cognitive impairment, it remains unclear whether plasma p-tau181, GFAP, and NfL offer independent predictive value beyond established clinical and imaging factors, and crucially, how these peripheral measures relate to central cognitive function.

Neuroimaging studies offer valuable mechanistic insights. In cognitive disorders, elevated levels of p-tau181, GFAP, and NfL, alongside a reduced Aβ42/40 ratio, have been correlated with patterns of brain atrophy (Oeckl et al., 2023; Sanchez et al., 2025). Specifically in PD, atrophy within limbic structures (e.g., hippocampus, entorhinal cortex, cingulate cortex), the basal ganglia, and generalized brain volume loss are associated with cognitive deficits (Mak et al., 2017; Zhou et al., 2020; Crowley et al., 2021). These regions are thus strong candidate mediators, potentially linking peripheral biomarker elevations to central cognitive manifestations. Despite this, whether atrophy in these specific regions mediates the association between plasma biomarkers and cognition in PD remains unestablished. Furthermore, whether cognitive status moderates this mediation pathway remains unknown.

To address these gaps, we employed an integrative approach combining plasma biomarkers, high-resolution structural magnetic resonance imaging (MRI), and cognitive assessment. Our study had three primary objectives: First, to evaluate the independent predictive value of plasma p-tau181, GFAP, NfL, and Aβ42/40 for global cognitive function in non-demented PD patients. Second, to test whether the influence of these plasma biomarkers on cognition is mediated by atrophy in *a priori* selected brain regions implicated in PD-related cognitive impairment, focusing on limbic and basal ganglia structures. Third, to examine whether the strength of this mediation pathway is moderated by cognitive status. Based on the established links between these biomarkers and neurodegenerative processes, we hypothesized that elevated plasma levels of p-tau181, GFAP, and NfL would be independently associated with worse cognitive performance, and that their effects would be mediated by regional brain atrophy. Furthermore, we explored whether these mediating effects differed between the two disease stages, namely, Parkinson’s disease with normal cognition (PD-NC) and PD with mild cognitive impairment (PD-MCI).

## 2 Materials and methods

### 2.1 Participants

This study included 89 PD patients and 40 healthy controls (HCs). All participants were right-handed to minimize the confounding influence of cerebral lateralization on structural measures. Patients were consecutively recruited from the movement disorders clinic at Wuhan First Hospital between January 2024 and June 2025. Diagnosis was confirmed by a movement disorder specialist according to the International Parkinson and Movement Disorder Society (MDS) clinical diagnostic criteria. HCs were community-dwelling volunteers matched to the patient group for age and sex. Exclusion criteria were as follows: age < 50 years; a diagnosis of dementia (meeting MDS criteria for PD-dementia or other standardized dementia criteria); inability to complete neuropsychological testing; major neurological or psychiatric diseases (e.g., stroke, epilepsy, brain tumor, major depressive disorder or anxiety disorder); current cognitive rehabilitation therapy; MRI contraindications; or poor-quality MRI data. All participants completed blood sampling for plasma biomarkers and routine laboratory tests, APOE genotyping, high-resolution structural MRI, and a standardized neuropsychological assessment. The mean interval between blood draw and MRI acquisition was 2.6 ± 1.1 days. The study was approved by the Ethics Committee of Wuhan First Hospital, and written informed consent was obtained from all participants.

### 2.2 Clinical, demographic, and laboratory assessments

#### 2.2.1 Clinical and demographic data

Demographic characteristics, medical history, and vascular risk factors were recorded for all participants, including age, sex, years of education, body mass index (BMI), and histories of hypertension, diabetes, and smoking. For PD patients, disease duration, age at motor onset, and motor symptom severity were documented. Motor severity was assessed using the Movement Disorder Society-Unified Parkinson’s Disease Rating Scale Part III (MDS-UPDRS-III) during the ON medication state. To ensure reliability, MDS-UPDRS-III assessments were performed by two experienced neurologists; any scoring discrepancies were resolved through consensus discussion.

#### 2.2.2 Routine laboratory tests

Fasting venous blood was collected from all participants in the morning using standard venipuncture. Serum fasting glucose and low-density lipoprotein cholesterol levels were measured. Plasma homocysteine was assayed from EDTA-anticoagulated samples. APOE ε4 genotyping was performed via polymerase chain reaction on genomic DNA, and participants carrying at least one ε4 allele were classified as APOE ε4 carriers.

#### 2.2.3 Plasma biomarker quantification

For plasma biomarker analysis, blood was collected into EDTA tubes. Plasma was separated by centrifugation (1,000 × *g*, 10 min, 4 °C), aliquoted, and stored at −80 °C until analysis. Concentrations of Aβ40, Aβ42, p-tau181, GFAP, and NfL were quantified using single-molecule array (Simoa) technology on an AST-Dx90 fully automated immunoassay analyzer (AstraBio, China) with commercially available Simoa kits. All assays were performed according to the manufacturer’s instructions. All samples were measured in duplicate, and the same kit lot number was used throughout. Samples were not subjected to repeated freeze-thaw cycles. The Aβ42/Aβ40 ratio was derived. Laboratory personnel were blinded to all clinical data.

### 2.3 Neuroimaging acquisition and processing

All participants underwent brain MRI on a 3.0T Siemens MAGNETOM Vida scanner using a 64-channel head-neck coil. Head motion was minimized with foam padding, and participants were instructed to remain still and awake with their eyes closed. High-resolution three-dimensional T1-weighted images were acquired using a magnetization-prepared rapid gradient-echo (MPRAGE) sequence (repetition time = 2200 ms, inversion time = 900 ms, echo time = 3.41 ms, flip angle = 8°, field of view = 240 × 240 mm^2^, matrix = 256 × 256, slice thickness = 0.94 mm, yielding an isotropic voxel size of 0.9 mm^3^). T2-weighted fluid-attenuated inversion recovery (FLAIR) images were also acquired for the assessment of white matter hyperintensities (repetition time = 7000 ms, inversion time = 2220 ms, echo time = 87 ms, flip angle = 120°, field of view = 215 × 230 mm^2^, slice thickness = 5 mm).

Cortical reconstruction and subcortical segmentation were performed using FreeSurfer (version 7.4.0). The standard pipeline included motion correction, non-brain tissue removal, intensity normalization, and automated parcellation. All segmentations were systematically visually inspected by an experienced neuroradiologist blinded to participant information; when necessary, minimal manual corrections were applied after adjusting parameters and re-running the corresponding steps. Regional volumes were extracted for the following structures: whole brain, lateral ventricles, hippocampus, entorhinal cortex, cingulate cortex, caudate nucleus, and putamen. Total intracranial volume (ICV) was extracted for normalization.

White matter hyperintensity (WMH) volume was automatically segmented from the T2-FLAIR images using the lesion segmentation toolbox in SPM12. WMH segmentations were similarly inspected by the same neuroradiologist, with minimal manual corrections applied when necessary. The absolute volume of each region and total WMH burden were divided by the ICV to obtain relative volumes (%) for statistical analysis, correcting for head size variation.

### 2.4 Cognitive assessment and classification

Cognitive function was assessed using both the Mini-Mental State Examination (MMSE) and the Montreal Cognitive Assessment (MoCA), with the MoCA serving as the primary tool for detailed cognitive profiling and classification. To minimize the potential confounding effect of motor and non-motor symptom fluctuations, the cognitive assessment was administered to all PD patients during the ON medication state. A trained neurologist, who was blinded to all participant biomarker and neuroimaging data, conducted and scored all cognitive tests. Cognitive status in PD was classified according to the Movement Disorder Society (MDS) Level II criteria for mild cognitive impairment. Based on MoCA subtest performance, we evaluated five cognitive domains: visuospatial/executive, naming, attention (digit span, vigilance, serial 7s), language (sentence repetition, verbal fluency), and delayed recall. PD-MCI was defined as performance more than 1.5 standard deviations below the healthy control mean in at least two domains. Patients not meeting this criterion were classified as PD-NC.

### 2.5 Statistical analysis

Statistical analyses were conducted using IBM SPSS Statistics (version 26.0) and the PROCESS macro. The normality of all continuous variables was assessed using the Shapiro-Wilk test. Accordingly, data are presented as mean ± standard deviation or median (interquartile range), and group comparisons were performed using parametric (*t*-test, ANOVA) or non-parametric (Mann-Whitney U, Kruskal-Wallis) tests as appropriate. For analyses involving more than two groups, *post hoc* pairwise comparisons were conducted with the Bonferroni correction. Categorical data are presented as frequency (percentage) and were compared using the chi-square test. The significance threshold for all descriptive and group comparison analyses was set at *p* < 0.05. Plasma concentrations of Aβ42/40, p-tau181, GFAP, NfL, as well as the volumetric measures of whole brain, lateral ventricles, putamen, and white matter hyperintensity (WMH), exhibited skewed distributions. These variables were therefore ln-transformed to approximate normality. Subsequently, all continuous variables were standardized to z-scores for subsequent parametric analyses.

Bivariate associations among plasma biomarkers, regional brain volumes, and MoCA scores were examined using Pearson correlation analysis on the standardized variables. To address collinearity among structural measures, a principal component analysis (PCA) was performed on the volumes of five key regions (hippocampus, entorhinal cortex, cingulate cortex, caudate, putamen), all normalized to ICV. The first component, explaining the greatest proportion of variance, was retained as a composite cognition-related atrophy factor.

Hierarchical multiple linear regression was used to evaluate the independent contribution of each plasma biomarker to the MoCA score. Covariates were entered sequentially across four base models: demographics (age, sex, education), then PD-specific features (disease duration, MDS-UPDRS-III), followed by vascular burden (WMH volume), and finally the composite atrophy factor derived from PCA. To isolate the unique effect of each biomarker, the standardized ln-transformed value of Aβ42/40, p-tau181, GFAP, or NfL was added individually to this final base model in separate analyses.

**FIGURE 1:**
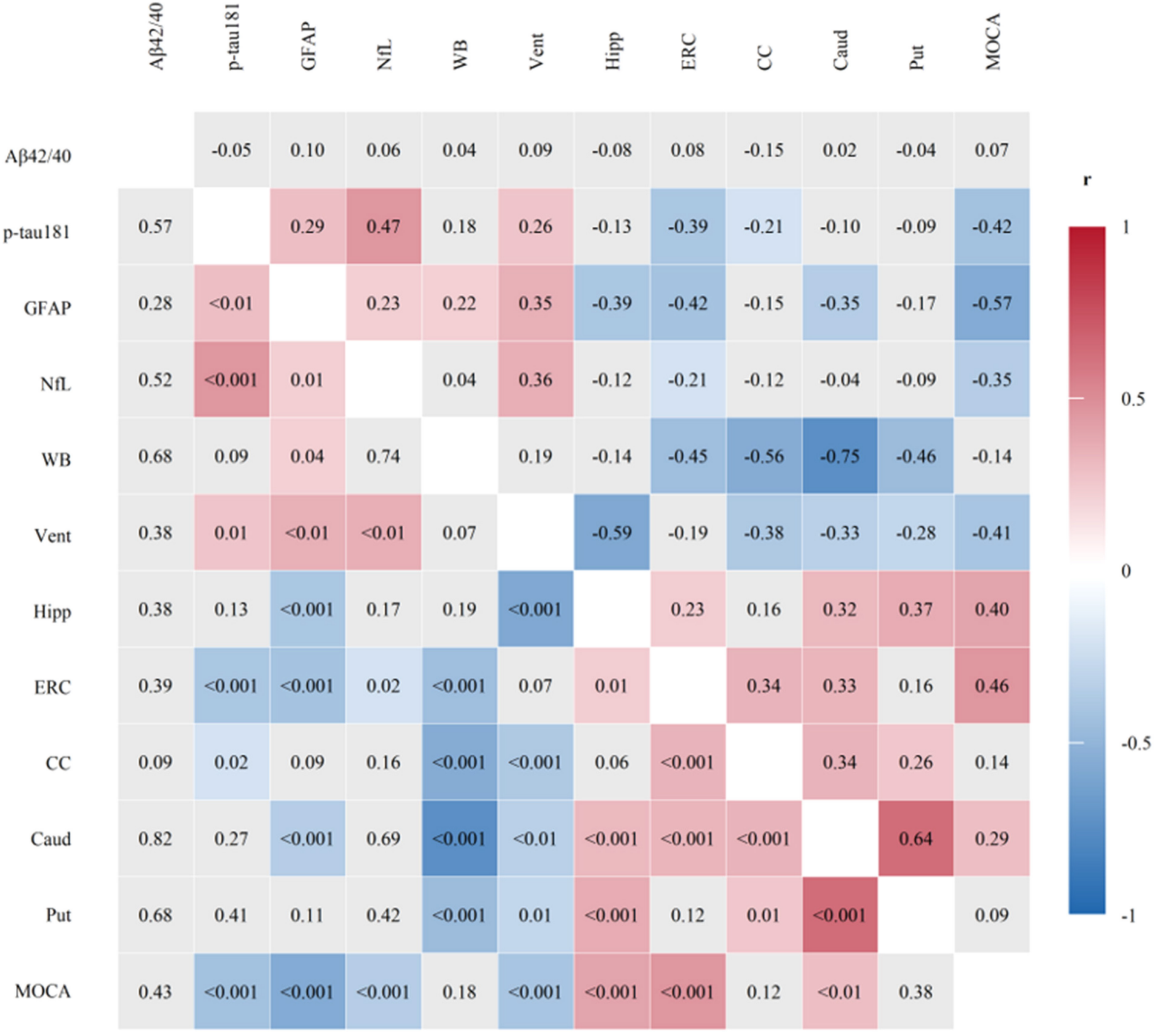
Correlation matrix of plasma biomarkers, key brain region volumes, and cognitive performance. Plasma biomarkers were ln-transformed, and all variables were standardized. Upper triangle: Pearson correlation coefficients (r). Lower triangle: *p*-values. Cells with *p* < 0.05 are colored by r (blue–white–red); non-significant cells are light gray. Brain region: WB, whole brain; Vent, ventricles; Hipp, hippocampus; ERC, entorhinal cortex; CC, cingulate cortex; Caud, caudate nucleus; Put, putamen.

While the composite factor was used in regression models to control for global brain atrophy, the mediation analysis (PROCESS Macro Model 4) tested whether individual regional volumes mediated the biomarker-cognition associations. Based on *a priori* anatomical relevance to PD-related cognitive circuits, as well as the group differences and bivariate correlations presented in Table 1 and Section “3.2 Plasma biomarkers, brain structure, and cognitive performance are interrelated” (Figure 1), we selected four ICV-normalized volumes–the entorhinal cortex, hippocampus, caudate, and lateral ventricles–as candidate mediators. All models were adjusted for age, sex, education, APOE ε4 status, disease duration, MDS-UPDRS-III, and WMH volume. For significant mediation pathways, moderated mediation analysis (PROCESS Macro Model 59) was conducted to examine whether the strength of mediation differed between PD-NC and PD-MCI groups. The significance of mediated effects in both mediation and moderated mediation analyses was evaluated using bias-corrected bootstrap confidence intervals (5000 resamples), with effects considered significant if the 95% CI did not contain zero. Given the hypothesis-driven selection of a small number of candidate mediators, we did not apply an additional correction for multiple comparisons.

**TABLE 1:** Baseline demographic, clinical, and neuroimaging characteristics.

Measure	HC (*N* = 40)	PD-NC (*N* = 37)	PD-MCI (*N* = 52)	Statistic	*P*-value
Demographics					
Age, years	67.40 ± 5.86	69.51 ± 7.62	70.83 ± 7.40	*F* = 2.70	0.07
Male, *n* (%)	22 (55.0)	24 (64.9)	20 (38.5)	χ^2^ = 6.37	0.04*
Education, years	9.00 (9.00, 12.00)	12.00 (9.00, 15.00)	9.00 (6.00, 12.00)	*H* = 10.74	<0.01**
BMI, kg/m^2^	23.52 ± 2.21	23.68 ± 2.51	22.45 ± 3.86	*F* = 1.85	0.16
PD disease features					
Onset age, years	–	66.00 (62.00, 69.50)	67.25 (63.00, 72.00)	*U* = 872.50	0.46
Disease duration, years	–	3.00 (2.00, 5.25)	4.25 (2.50, 6.00)	*U* = 786.50	0.14
MDS-UPDRS-III	–	29.70 ± 8.87	35.90 ± 10.23	*t* = −3.05	<0.01**
Clinical features					
Hypertension, *n* (%)	18 (45.0)	16 (43.2)	27 (51.9)	χ^2^ = 0.78	0.68
Diabetes, *n* (%)	10 (25.0)	10 (27.0)	17 (32.7)	χ^2^ = 0.72	0.70
Smoking, packs/year	0.00 (0.00, 13.75)	0.00 (0.00, 20.00)	0.00 (0.00, 10.00)	*H* = 1.36	0.51
APOE ε4 carrier, *n* (%)	7 (17.5)	5 (13.5)	20 (38.5)	χ^2^ = 8.87	0.01*
Laboratory tests					
Fasting glucose, mmol/L	5.42 (4.74, 6.16)	5.07 (4.55, 5.47)	5.20 (4.90, 6.31)	*H* = 5.25	0.07
Low-density lipoprotein cholesterol, mmol/L	3.13 ± 0.72	2.68 ± 0.83	2.76 ± 0.64	*F* = 4.35	0.02*
Homocysteine, μmol/L	13.35 (10.00, 16.28)	14.40 (12.00, 21.15)	15.05 (11.40, 18.35)	*H* = 3.00	0.22
Aβ42/40	0.15 (0.12, 0.24)	0.21 (0.16, 0.26)	0.19 (0.11, 0.40)	*H* = 1.36	0.51
GFAP, pg/mL	5.51 (3.73, 8.23)	13.06 (5.97, 20.07)	22.37 (15.48, 34.12)	*H* = 58.90	<0.001***
NfL, pg/mL	3.07 (2.04, 5.61)	1.53 (0.32, 3.39)	6.60 (2.01, 12.59)	*H* = 19.65	<0.001***
p-tau181, pg/mL	2.28 (1.80, 2.82)	0.83 (0.33, 2.44)	3.29 (0.78, 7.43)	*H* = 15.65	<0.001***
MRI volumes					
Whole brain, %	88.97 (86.58, 90.56)	86.17 (85.35, 89.90)	86.60 (85.13, 90.00)	*H* = 6.98	0.03*
Ventricles, %	2.20 (1.97, 2.44)	2.66 (2.30, 2.91)	3.20 (2.93, 3.69)	*H* = 17.66	<0.001***
Hippocampus, %	0.51 ± 0.07	0.46 ± 0.10	0.41 ± 0.11	*F* = 11.54	<0.001***
Entorhinal cortex, %	0.26 ± 0.04	0.26 ± 0.04	0.23 ± 0.05	*F* = 10.00	<0.001***
Cingulate cortex, %	0.86 ± 0.10	0.85 ± 0.12	0.84 ± 0.13	*F* = 0.35	0.71
Caudate, %	0.47 (0.42, 0.51)	0.41 (0.32, 0.49)	0.43 (0.32, 0.48)	*H* = 7.81	0.02*
Putamen, %	0.65 (0.52, 0.71)	0.62 (0.58, 0.67)	0.62 (0.52, 0.69)	*H* = 0.65	0.72
WMH, %	0.39 (0.30, 0.76)	0.66 (0.39, 1.14)	1.08 (0.66, 1.98)	*H* = 19.72	<0.001***
Cognitive scores					
MMSE	29.00 (29.00, 30.00)	28.00 (27.00, 29.00)	24.00 (18.00, 26.00)	*H* = 71.67	<0.001***
MOCA	27.00 (25.25, 28.00)	26.00 (26.00, 27.00)	20.00 (15.00, 23.00)	*H* = 84.59	<0.001***

Data are presented as mean ± standard deviation for normally distributed variables, median (interquartile range) for non-normally distributed variables, or *n* (%). MDS-UPDRS-III, Movement Disorder Society Unified Parkinson’s Disease Rating Scale Part III; WMH, white matter hyperintensities; MMSE, Mini-Mental State Examination. All imaging measures are adjusted for total intracranial volume. Group comparisons used one-way ANOVA (F) for normally distributed variables, Kruskal-Wallis H test (H) for non-normally distributed variables, or chi-square test (χ^2^) for categorical variables. *p < 0.05, **p < 0.01, ***p < 0.001.

## 3 Results

### 3.1 Participant characteristics and group differences

We enrolled 129 participants, including 40 HCs, 37 PD-NC, and 52 PD-MCI. Their baseline characteristics are detailed in Table 1. As intended by the cohort design, robust differences in cognitive performance were confirmed. Both the MMSE and the MoCA scores differed significantly across groups (all *p* < 0.001), with the PD-MCI group scoring lowest, followed by the PD-NC and HC groups. Consistent with profiles of progressive impairment, the PD-MCI group included a lower proportion of males, had fewer years of education, a higher APOE ε4 carrier rate, and more severe motor symptoms (higher MDS-UPDRS-III scores) compared to the PD-NC group (all *p* < 0.05).

At baseline, the PD-MCI group exhibited a distinct biomarker and neuroimaging profile. Plasma levels of GFAP, NfL, and p-tau181 were significantly elevated in the PD-MCI group compared to both the PD-NC and HC groups (all *p* < 0.001); while the Aβ42/40 ratio did not differ significantly. Structurally, the PD-MCI group exhibited significant atrophy in the hippocampus and entorhinal cortex, greater ventricular enlargement, and a higher burden of white matter hyperintensities compared to the other groups (all *p* < 0.001). Volumes of the cingulate cortex and putamen were comparable across groups. The three groups did not differ significantly in age, vascular risk factors, and the volumes of the cingulate cortex and putamen. All subsequent analyses controlled for the confounding variables of sex, education, APOE ε4 status, and MDS-UPDRS-III score.

### 3.2 Plasma biomarkers, brain structure, and cognitive performance are interrelated

We first examined the bivariate relationships between plasma biomarkers, regional brain volumes, and cognitive performance. A correlation matrix of the standardized variables is presented in Figure 1. As hypothesized, plasma levels of p-tau181, GFAP, and NfL each showed significant negative correlations with the MoCA score (*r* = −0.43, −0.57, and −0.35; all *p* < 0.001). In contrast, the plasma Aβ42/40 ratio was not associated with cognitive performance.

Regarding brain structure, higher levels of p-tau181 and GFAP were specifically associated with lower volume of entorhinal cortex (ERC) (*r* = −0.39 and −0.42; all *p* < 0.001). Both GFAP and NfL levels correlated with ventricular enlargement (*r* = 0.35 and 0.36; *p* = 0.001). The ERC volume was positively correlated with MoCA scores (*r* = 0.46, *p* < 0.001), while ventricular volume was negatively correlated (*r* = −0.41, *p* < 0.001). No significant correlations were observed for the Aβ42/40 ratio with any brain structural measure.

### 3.3 Derivation of a composite brain atrophy factor

To mitigate multicollinearity among the five key regional brain volumes (hippocampus, entorhinal cortex, cingulate cortex, caudate, and putamen) and to derive a composite index of cognition-related atrophy, we performed a principal component analysis (PCA). The analysis yielded one dominant component with an eigenvalue > 1, which explained 46.1% of the total variance (KMO = 0.66; Bartlett’s test *p* < 0.001). As shown in Table 2, this component had positive loadings from all five regions, indicating that atrophy across these structures occurs in a coordinated manner. We therefore termed this component the cognitive-related brain atrophy factor and used it as a covariate representing global structural integrity in subsequent regression models. Specifically, its inclusion was intended to control for overall brain atrophy and to reduce multicollinearity among the individual regional volumes, thereby allowing a more stringent evaluation of the independent contributions of plasma biomarkers.

**TABLE 2:** Principal component analysis loadings for the cognitive-related brain atrophy factor.

Brain region	Components	Communality
Caudate	0.85	0.73
Putamen	0.76	0.58
Cingulate cortex	0.64	0.41
Entorhinal cortex	0.55	0.30
Hippocampus	0.53	0.28
Variance explained	46.10%	

### 3.4 Plasma biomarkers independently predict cognitive performance

To determine whether plasma biomarkers predict cognitive function independently of established factors, we conducted hierarchical multiple regression with MoCA score as the dependent variable (Table 3). The base model (Model 4), which included demographics (age, sex, education), PD-specific features (disease duration, MDS-UPDRS-III), vascular burden (WMH volume), and the composite cognitive-related brain atrophy factor, explained 59.8% of the variance in MoCA scores.

**TABLE 3:** Hierarchical regression analysis assessing the independent contribution of plasma biomarkers to MoCA scores.

Predictors	Model 1	Model 2	Model 3	Model 4	Model 5a (Aβ42/40)	Model 5b (p-tau181)	Model 5c (GFAP)	Model 5d (NfL)
Age	−0.27**	−0.18	−0.06	−0.02	−0.01	−0.04	−0.01	0.01
Sex	0.17	0.14	0.17	0.14	0.13	0.11	0.13	0.11
Education	0.30**	0.28**	0.25**	0.27***	0.28**	0.24**	0.26**	0.26**
APOE ε4	−0.41***	−0.35***	−0.33***	−0.26**	−0.26**	−0.25**	−0.24**	−0.26**
Disease duration	–	0.02	−0.03	−0.03	−0.03	−0.05	−0.03	−0.04
MDS-UPDRS-III	–	−0.34***	−0.34***	−0.38***	−0.38***	−0.32***	−0.33***	−0.36***
WMH	–	–	−0.24**	−0.18*	−0.18*	−0.12	−0.17*	−0.13
Cognitive-related brain atrophy factor	–	–	–	0.24**	0.24**	0.18*	0.19*	0.21**
Aβ42/40	–	–	–	–	0.03	–	–	–
p-tau181	–	–	–	–	–	−0.22**	–	–
GFAP	–	–	–	–	–	–	−0.17*	–
NfL	–	–	–	–	–	–	–	−0.20*
Model statistics								
R^2^ (%)	41.20	51.00	55.20	59.80	59.90	63.40	61.70	63.00
Adjusted R^2^ (%)	38.40	47.40	51.30	55.80	55.30	59.20	57.40	58.70
ΔR^2^ (%)	–	9.80***	4.20**	4.60**	0.10	3.60**	1.90*	3.20*

Table shows standardized beta coefficients (β). Model build order: Model 1 (demographics); Model 2 (Model 1 + PD features); Model 3 (Model 2 + WMH); Model 4 (Model 3 + composite cognitive-related brain atrophy factor); Models 5a–d (Model 4 + individual plasma biomarker). WMH, white matter hyperintensities. *p < 0.05, **p < 0.01, ***p < 0.001.

When added individually to this comprehensive model, plasma p-tau181, GFAP, and NfL each provided significant incremental predictive value. p-tau181 accounted for the largest incremental variance (ΔR^2^ = 3.6%, β = −0.22, *p* = 0.007), followed by NfL (ΔR^2^ = 3.2%, β = −0.20, *p* = 0.022) and GFAP (ΔR^2^ = 1.9%, β = −0.17, *p* = 0.036). Notably, the inclusion of each biomarker attenuated the regression coefficient of the composite atrophy factor, suggesting that part of its association with cognition is shared with these plasma measures. In contrast, the plasma Aβ42/40 ratio showed no independent association with MoCA scores (β = 0.03, *p* = 0.690).

### 3.5 ERC atrophy mediates the effects of plasma biomarkers on cognition

**FIGURE 2:**
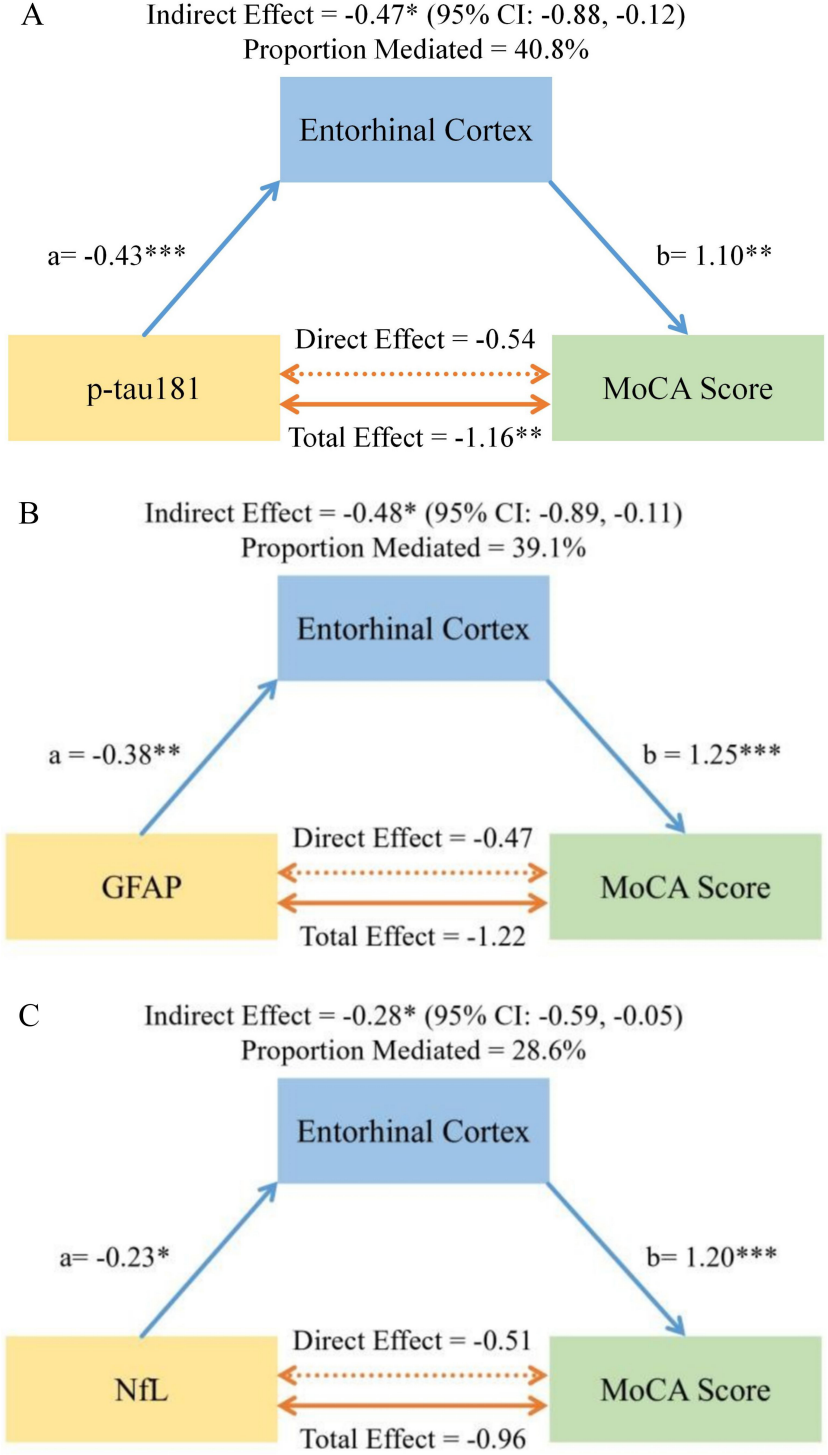
Path diagrams show the mediating role of entorhinal cortex volume in the relationship of plasma **(A)** p-tau181, **(B)** GFAP, and **(C)** NfL with MoCA score. Path coefficients are standardized estimates (path a: biomarker to EC; path b: EC to MoCA). Total, direct, and indirect effects are presented with 95% confidence intervals. All models were adjusted for age, sex, education, APOE ε4 carrier status, disease duration, MDS-UPDRS-III score, and white matter hyperintensity (WMH) volume. *p < 0.05, **p < 0.01, ***p < 0.001.

We next tested whether the associations of p-tau181, GFAP, and NfL with cognitive performance were mediated by specific brain structures, using parallel multiple mediation models adjusted for the covariates detailed in the section “2.5 Statistical analysis” (Figure 2). The ERC emerged as the sole significant mediator for all three biomarkers.

The indirect effect of p-tau181 on MoCA through ERC atrophy was significant (β = −0.47, 95% CI: −0.91 to −0.13), accounting for 40.8% of its total effect. For GFAP, the indirect effect via ERC was also significant (β = −0.48, 95% CI: −0.94 to −0.14; 39.1% of total effect), with a trend toward an additional indirect effect through ventricular enlargement (β = −0.24, 95% CI: −0.66 to 0.03). For NfL, the indirect effect through ERC atrophy was significant (β = −0.28, 95% CI: −0.61 to −0.04), accounting for 28.6% of the total effect. After accounting for this mediation, the direct paths from each biomarker to cognition were non-significant. No significant mediating effects were observed for the hippocampus, caudate nucleus, or lateral ventricles. Complete mediation results for all four tested regions are provided in Supplementary Table 1.

### 3.6 Cognitive status moderates the link between plasma biomarkers and ERC atrophy

**FIGURE 3:**
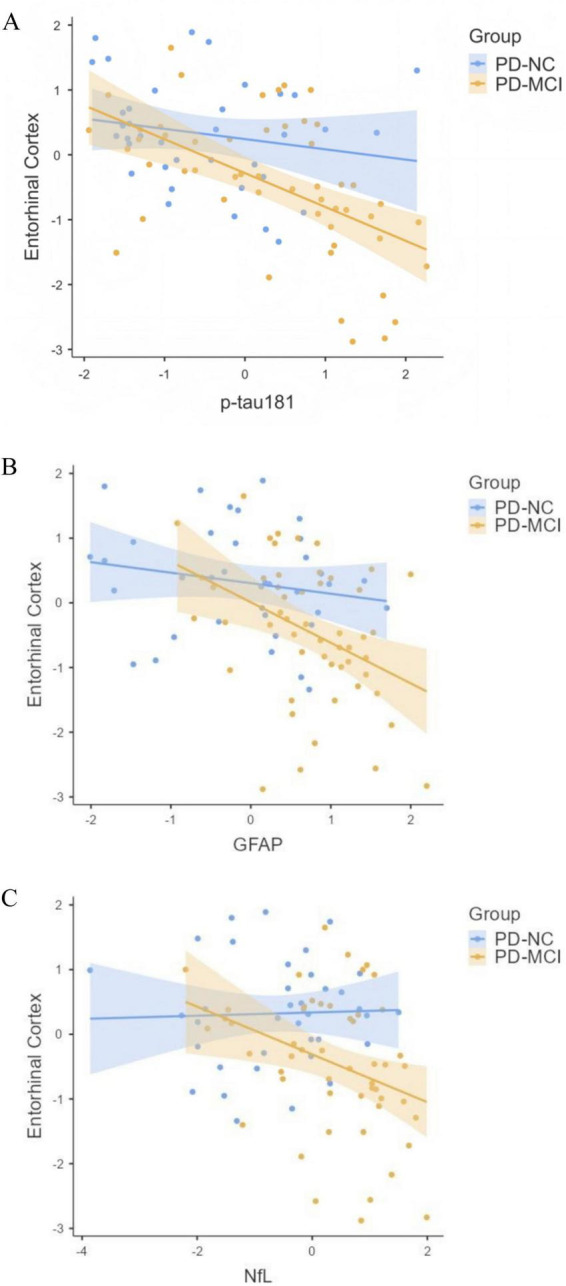
Cognitive status moderates the association between plasma biomarkers and entorhinal cortex volume. The figure shows the associations between standardized plasma **(A)** p-tau181, **(B)** GFAP, and **(C)** NfL with standardized ERC volume, stratified by cognitive status (PD-NC vs. PD-MCI). The relationships are derived from the moderated mediation model (PROCESS Model 59). Regression parameters: p-tau181 (PD-NC: β = –0.17, *p* = 0.28; PD-MCI: β = –0.55, *p* < 0.001); GFAP (PD-NC: β = –0.12, *p* = 0.49; PD-MCI: β = –0.58, *p* = 0.01); NfL (PD-NC: β = 0.05, *p* = 0.74; PD-MCI: β = –0.40, *p* = 0.01). Full statistics are provided in Supplementary Table 2.

We examined whether the mediation pathway differed between PD-NC and PD-MCI patients using moderated mediation analysis (Figure 3). Cognitive status significantly moderated the association between plasma biomarkers and ERC volume for both p-tau181 (β = −0.39, *p* = 0.04) and NfL (β = −0.44, *p* = 0.03), with a strong trend for GFAP (β = −0.46, *p* = 0.08). Probing these interactions revealed that the negative associations between each biomarker and ERC volume were significant only within the PD-MCI group (all *p* < 0.01), not in the PD-NC group.

Consequently, the conditional indirect effect of NfL on cognition through ERC atrophy was significant only in the PD-MCI group (β = −0.43, 95% CI: −0.98 to −0.03), and the index of moderated mediation was statistically significant (Index = −0.44, 95% CI: −1.02 to −0.03). For p-tau181 and GFAP, the indirect effects in the PD-MCI group were marginally significant, but the moderated mediation indices did not reach significance.

## 4 Discussion

This study identifies a potential pathway linking peripheral pathology to cognitive decline in Parkinson’s disease. We demonstrate that plasma p-tau181, GFAP, and NfL–markers of tau phosphorylation, astrocytic reactivity, and axonal injury, respectively–each independently predict global cognitive impairment in non-demented PD. Crucially, their deleterious associations converge on a common neural correlate: atrophy of ERC. Furthermore, the pathway linking NfL to cognition through entorhinal atrophy is not static but dynamically strengthened with disease progression, becoming significantly stronger upon transition to PD-MCI. These findings position ERC as a critical integrative node where multiple pathogenic processes translate into cognitive dysfunction and highlight NfL as a potential biomarker for tracking progression into clinically significant cognitive impairment.

### 4.1 Plasma biomarkers reflect distinct pathogenic processes in PD cognitive impairment

Our analyses establish that p-tau181, GFAP, and NfL independently predict cognitive impairment in PD, after rigorously controlling for demographics, motor severity, vascular burden, and overall brain atrophy. The lack of significant interactions among them suggests they capture distinct, complementary pathological processes in PD cognitive impairment.

Plasma p-tau181 exhibited the strongest independent association with global cognitive performance. Its elevation in PD may reflect co-aggregating tau pathology, which is known to interact synergistically with α-synuclein to worsen clinical outcomes (Li and Li, 2024; Franzmeier et al., 2025; Hu et al., 2025). Thus, elevated p-tau181 could help identify a PD subtype characterized by a more aggressive, mixed proteinopathy. GFAP also independently contributed, reinforcing the role of neuroinflammation in PD-related cognitive impairment. Its trend-level association with ventricular enlargement further suggests a link between neuroinflammation and broader brain tissue loss (Li et al., 2025; Popov et al., 2023; Liu et al., 2023). The predictive value of NfL confirms that axonal injury is a central final common pathway to functional decline (Khalil et al., 2018). Together, these biomarkers provide a more granular framework for stratifying cognitive risk in PD than any single measure.

### 4.2 ERC atrophy integrates multiple pathological processes

Atrophy of ERC commonly mediated the detrimental effects of plasma p-tau181, GFAP, and NfL on cognition, identifying it as a critical convergent point in PD (Jia et al., 2019; Goldman et al., 2012). The unique vulnerability of ERC to these disparate biomarkers is anatomically and physiologically grounded (Zhang et al., 2024). The ERC is an early site for tau deposition and a hub for its trans-neuronal spread, which may explain its strong association with p-tau181 and its susceptibility to tau-induced neuronal dysfunction (Nassif et al., 2022). Concurrently, its position as a major glymphatic conduit and metabolically active region makes it vulnerable to inflammatory processes and astrocytic dysfunction, captured by GFAP (Pociūtė et al., 2024). The dense white matter projections of the ERC also render it acutely sensitive to axonal injury, reflected by NfL levels (Hu et al., 2025). Thus, ERC atrophy integrates and reflects the combined effects of pathological protein deposition, neuroinflammation, and axonal injury. Notably, the lack of significant mediation via basal ganglia structures suggests that, in the context of our plasma biomarkers and global cognitive measure, limbic pathways (particularly the ERC) may play a more prominent mediating role. However, this finding does not imply independence between cognitive and motor circuits, nor does it exclude contributions from frontostriatal networks. The absence of significant mediation may also reflect limited statistical power or the specific cognitive domains assessed. Turning to limbic structures, the absence of mediation via the hippocampus, despite its established role in PD cognition, suggests that ERC atrophy may represent a more convergent upstream event, with hippocampal changes potentially occurring downstream or being influenced by other factors not captured by our plasma biomarkers.

### 4.3 NfL as a dynamic, stage-specific biomarker

The mediating pathway linking plasma NfL to cognition through ERC atrophy is not static but is significantly moderated by disease stage. This pathway was markedly stronger and achieved full mediation specifically in the PD-MCI group, but was non-significant in the PD-NC group. This finding suggests that the association between axonal injury and brain region integrity changes with disease stage. One possible interpretation is that in the PD-NC stage, the association between axonal injury (reflected by NfL) and ERC atrophy may be below the detection threshold or buffered by neural reserve, resulting in a non-significant mediation pathway (Montine et al., 2019; Carapelle et al., 2020; Corbo et al., 2023). During this phase, NfL may serve as a biomarker of generalized, subclinical neurodegenerative burden. With progression to the PD-MCI stage, cumulative axonal damage may reach a level where its association with ERC atrophy becomes detectable, thereby unmasking a significant mediation pathway. An alternative interpretation is that the biological relevance of NfL changes with disease stage (Goldman et al., 2012; Hu et al., 2025). We acknowledge that these interpretations are *post hoc* and speculative; future longitudinal studies are needed to clarify the temporal dynamics. Thus, serial assessment of plasma NfL, particularly when combined with monitoring of ERC structure, could help identify PD patients at a preclinical stage who are at the highest risk for imminent cognitive decline. This positions NfL not merely as a prognostic marker, but as a potential tool for stratifying patients for early interventions aimed at cognitive preservation.

### 4.4 Limitations and future directions

This study has limitations. Its cross-sectional nature cannot establish causality. Although our mediation models propose a directional pathway, this temporal sequence remains hypothetical and requires validation in longitudinal studies. The reverse direction–that ERC atrophy contributes to biomarker elevation–cannot be ruled out given the cross-sectional design. Additionally, the single-center sample may limit generalizability, and the sample size–particularly for the moderated mediation analyses–is relatively modest, which may have limited statistical power for detecting such effects. Furthermore, our selection of candidate brain regions for mediation analysis, while anatomically motivated and empirically informed, remains exploratory. The possibility that other cortical or subcortical regions not examined here could also mediate these associations cannot be excluded. Finally, the PD-MCI group had a higher proportion of APOE ε4 carriers and greater WMH burden. Although we adjusted for both, we cannot exclude mixed Alzheimer-related or vascular pathology. Moreover, the lack of molecular imaging precludes direct linkage between plasma markers and central pathology. Future longitudinal, multi-center studies are needed to validate the temporal sequence of these biomarkers and ERC atrophy. Ultimately, this multi-modal biomarker approach should be tested for its utility in stratifying patients for clinical trials targeting cognitive impairment in PD.

## 5 Conclusion

Plasma p-tau181, GFAP, and NfL are associated with cognitive impairment in Parkinson’s disease via ERC atrophy. The NfL pathway is selectively enhanced in mild cognitive impairment, highlighting its role as a dynamic, stage-specific biomarker. Integrated plasma and ERC imaging can refine stratification for cognitive impairment in PD.

## Data Availability

The raw data supporting the conclusions of this article will be made available by the authors, without undue reservation.
