# Supplementary material for: Entorhinal cortex atrophy mediates the association of plasma p-tau181, GFAP, and NfL with cognitive impairment in Parkinson’s disease
Source: Front Aging Neurosci. 2026 May 8;18:1791756. doi: 10.3389/fnagi.2026.1791756 (PMC13194581; doi:10.3389/fnagi.2026.1791756)
Supplement: Supplementary file 1 [file Data_Sheet_1.docx]

**Table S1.** Indirect effects of plasma biomarkers on MoCA scores through candidate brain regions.

| **Mediator** | **Biomarker** | **Indirect effect (β)** | **Boot SE** | **95% Bootstrap CI** |
| --- | --- | --- | --- | --- |
| entorhinal cortex | p-tau181 | -0.47 | 0.19 | (-0.88, -0.12) |
| entorhinal cortex | GFAP | -0.48 | 0.20 | (-0.89, -0.11) |
| entorhinal cortex | NfL | -0.28 | 0.14 | (-0.59, -0.05) |
| Hippocampus | p-tau181 | -0.05 | 0.07 | (-0.22, 0.07) |
| Hippocampus | GFAP | -0.10 | 0.10 | (-0.30, 0.11) |
| Hippocampus | NfL | -0.03 | 0.06 | (-0.18, 0.08) |
| Caudate | p-tau181 | 0.01 | 0.07 | (-0.15, 0.15) |
| Caudate | GFAP | 0.07 | 0.16 | (-0.24, 0.42) |
| Caudate | NfL | 0.00 | 0.05 | (-0.10, 0.12) |
| Ventricle | p-tau181 | -0.11 | 0.12 | (-0.42, 0.05) |
| Ventricle | GFAP | -0.24 | 0.18 | (-0.66, 0.03) |
| Ventricle | NfL | -0.14 | 0.12 | (-0.44, 0.04) |

Indirect effects (β) and 95% bootstrap CIs were estimated using PROCESS Macro Model 4 with 5,000 resamples. An effect is considered significant if the 95% CI does not include zero. Results for the entorhinal cortex are also presented in Figure 2 of the main manuscript. CI, confidence interval; Boot SE, bootstrap standard error.

**Table S2.** Conditional effects of plasma biomarkers on entorhinal cortex volume stratified by cognitive status, and interaction terms.

| **Biomarker** | **Cognitive group** | **Effect (slope)** | **SE** | **p-value** | **95% CI** |
| --- | --- | --- | --- | --- | --- |
| Conditional effects |  |  |  |  |  |
| p-tau181 | PD-NC | -0.17 | 0.15 | 0.28 | (-0.47, 0.14) |
| p-tau181 | PD-MCI | -0.55 | 0.12 | <0.001 | (-0.79, -0.32) |
| GFAP | PD-NC | -0.12 | 0.17 | 0.49 | (-0.47, 0.23) |
| GFAP | PD-MCI | -0.58 | 0.20 | 0.01 | (-0.98, -0.18) |
| NfL | PD-NC | 0.05 | 0.14 | 0.74 | (-0.24, 0.33) |
| NfL | PD-MCI | -0.40 | 0.14 | 0.01 | (-0.69, -0.11) |
| Interaction terms |  | β | SE | p-value | 95% CI |
| p-tau181 × Cognitive status |  | -0.39 | 0.19 | 0.04 | (-0.77, -0.002) |
| GFAP × Cognitive status |  | -0.46 | 0.26 | 0.08 | (-0.98, 0.06) |
| NfL × Cognitive status |  | -0.44 | 0.20 | 0.03 | (-0.85, -0.04) |

Effects are unstandardized regression coefficients. CI, confidence interval; Boot SE, bootstrap standard error. PD‑NC, Parkinson's disease with normal cognition; PD‑MCI, Parkinson's disease with mild cognitive impairment.
